# Student Engagement as a General Factor of Classroom Experience: Associations with Student Practices and Educational Outcomes in a University Gateway Course

**DOI:** 10.3389/fpsyg.2017.00994

**Published:** 2017-06-15

**Authors:** David J. Shernof, Erik A. Ruzek, Alexander J. Sannella, Roberta Y. Schorr, Lina Sanchez-Wall, Denise M. Bressler

**Affiliations:** ^1^Center for Mathematics, Science, and Computer Education, Department of School Psychology, Graduate School of Applied and Professional Psychology, Rutgers University, The State University of New JerseyPiscataway, NJ, United States; ^2^Center for Advanced Study of Teaching and Learning, University of VirginiaCharlottesville, VA, United States; ^3^Department of Accounting and Information Systems, Rutgers Business School – Newark and New BrunswickNewark, NJ, United States; ^4^Department of Urban Education, Rutgers University, The State University of New JerseyNewark, NJ, United States; ^5^Peter Sammartino School of Education, Fairleigh Dickinson UniversityTeaneck, NJ, United States; ^6^Center for Mathematics, Science, and Computer Education, Rutgers University, The State University of New JerseyPiscataway, NJ, United States

**Keywords:** student, engagement, classroom, MSEM, ESM, bifactor, university, learning

## Abstract

The purpose of this study was to evaluate a model for considering general and specific elements of student experience in a gateway course in undergraduate Financial Accounting in a large university on the East Coast, USA. Specifically, the study evaluated a bifactor analytic strategy including a general factor of student classroom experience, conceptualized as student engagement as rooted in flow theory, as well as factors representing specific dimensions of experience. The study further evaluated the association between these general and specific factors and both student classroom practices and educational outcomes. The sample of students (*N* = 407) in two cohorts of the undergraduate financial accounting course participated in the Experience Sampling Method (ESM) measuring students' classroom practices, perceptions, engagement, and perceived learning throughout the one-semester course. Course grade information was also collected. Results showed that a two-level bifactor model fit the data better than two traditional (i.e., non-bifactor) models and also avoided significant multicollinearity of the traditional models. In addition to student engagement (general factor), specific dimensions of classroom experience in the bifactor model at the within-student level included intrinsic motivation, academic intensity, salience, and classroom self-esteem. At the between-student level, specific aspects included work orientation, learning orientation, classroom self-esteem, and disengagement. Multilevel Structural Equation Modeling (MSEM) demonstrated that sitting in the front of the classroom (compared to the sitting in the back), taking notes, active listening, and working on problems during class had a positive effect on within-student variation in student engagement and attention. Engagement, in turn, predicted perceived learning. With respect to between-student effects, the tendency to sit in front seats had a significant effect on student engagement, which in turn had a significant effect on perceived learning and course grades. A significant indirect relationship of seating and active learning strategies on learning and course grade as mediated by student engagement was found. Support for the general aspect of student classroom experience was interpreted with flow theory and suggested the need for additional research. Findings also suggested that active learning strategies are associated with positive learning outcomes even in educational environments where possibilities for action are relatively constrained.

## Introduction

Cognitive, emotional, and social forms of student engagement are important resources that students can draw on in order to perform well in university courses, including large lecture courses (Kuh et al., 2008; Svanum and Bigatti, 2009). This conclusion have been derived consistently from a decade of research, including large-scale surveys of student engagement completed annually at hundreds of institutions of higher education in the United States (most recently, the National Survey of Student Engagement, 2016). Research studies suggest that student engagement during the first few years of university coursework is particularly important (Ketonen et al., 2016). This is especially true in required gateway courses such as college-level algebra, introduction to economics, and introduction to financial accounting. For such courses, failure or low performance can lead to withdrawal and dropout. Based on their level of engagement in such courses, students may also be encouraged or discouraged to take more advanced coursework in a discipline, affecting choice of major. Students' perception of their own engagement can also impact career choices and commitments (Grier-Reed et al., 2012).

These studies and many others across grades K-12 provide credible evidence of the value of multiple forms of student engagement for learning, school identification, academic performance, and school completion (Wang and Holcombe, 2010; Reyes et al., 2012; Rumberger and Rotermund, 2012; Voelkl, 2012; Lee, 2014). Nevertheless, we know relatively little about how students' classroom practices and perceptions from one class period to another influence levels of engagement and experiences in gateway courses taken in the first several years of university enrollment, and how those classroom experiences influence educational outcomes such as learning and course performance. Prior research also suggests a number of important conceptual, methodological, and analytic questions regarding student engagement (Eccles and Wang, 2012; Reschly and Christenson, 2012; Eccles, 2016; Fredricks et al., 2016). This includes definitions, conceptualizations, and measurements of student engagement (Finn and Zimmer, 2012). One issue of focus in our study relates to the dimensionality of student engagement, including the possibility that it may encompass a general or unidimensional aspect of classroom experience. We investigated this issue in the context of a study of student engagement in undergraduate Financial Accounting, a gateway course for students interested in pursuing accounting or related majors, with primarily freshman and sophomore enrollment.

Another area in need of further clarification pertains to how student engagement and other more specific dimensions of classroom experience may relate to course learning and performance. Although the research referenced above suggests a positive relationship among student engagement, learning, and course performance, there are challenges in parsing out multiple dimensions of engagement, and how they may relate to student outcomes. For example, concentration may be heightened by perceptions that instruction is challenging and relevant (i.e., the presence of academic press or intensity), having a pronounced influence on attention and short-term performance; whereas interest, enjoyment, and intrinsic motivation may be facilitated by perceptions of autonomy and competence (e.g., positive emotional response), influencing long-term commitment and outcomes (Shernoff et al., 2003; Shernoff, 2013).

In this study, we propose and evaluate a model for considering general and specific aspects of students' classroom experience, and their potential associations with precursors and outcomes of their experience. Theoretically, this model is based on flow theory (Csikszentmihalyi, 1990); and methodologically, it is rooted in instruments designed to examine flow experiences. Analytically, the model relies on a bifactor modeling strategy, which generates uncorrelated specific and general factors. These factors can be included in the same model, each examined as unique outcomes of students' classroom practices and perceptions, and as unique predictors of student outcomes such as learning and course performance (Chen et al., 2012).

Relatedly, how much of the effect of student engagement on learning and performance outcomes is a function of classroom effects versus person-level influences remains relatively unknown, especially in college classrooms. That is, students may come to class with different levels of achievement motivation that contribute toward more or less engaging or participatory behaviors in class, and they may be more or less emotionally engaged as the result of instructional or environmental features. To help parse out these influences, we utilized a multilevel approach that partitions within-student and between-student variance in engagement, student classroom practices, and perceived learning (Raudenbush and Bryk, 2002). Within-student variation in engagement can be conceptualized in terms of engagement as a state that may fluctuate by mood or context. Differences between students in average engagement can be conceptualized as a trait, including personality characteristics that may influence experience (Shernoff, 2013).

This work is situated within the larger conversation regarding the nature of student engagement. There is increasing agreement that student engagement is a multidimensional metaconstruct (Martin, 2007; Appleton et al., 2008; LaNasa et al., 2009; Fredricks, 2011; Goldin et al., 2011; Wang et al., 2011; Shernoff, 2013; Wang and Eccles, 2013; Hospel and Galand, 2016), and that its dimensions include behavioral, emotional, cognitive subtypes (Fredricks et al., 2004). This taxonomy was based, in large part, on the various ways that engagement has been measured within the field. For example, *behavioral engagement* is based on observational measures of how engrossed students are in school tasks, and the consistency of effort, participation, attendance, or good behavior typical of good students (Finn and Voelkl, 1993; Marks, 2000; Green et al., 2008). *Cognitive engagement* is usually measured as students' investment in learning, depth of processing, quality of thinking, or the mastering of concepts and skills (Blumenfeld, 1992; Newmann, 1992; Newmann and Wehlage, 1993); students' intrinsic motivation to learn (Brophy, 1987; Covington, 2000; Ryan and Deci, 2000; Sansone and Harackiewicz, 2000); and/or the use of self-regulated metacognitive strategies (Zimmerman, 1990). *Emotional engagement* refers to students' affect and emotions in schools, and includes measures of interest, boredom, happiness, sadness, and anxiety (Finn, 1989; Voelkl, 1997; Shernoff et al., 2003).

It is important to recognize that these subtypes provided, primarily, a *taxonomy* of engagement, usefully organizing the great variety of conceptualizations and measurements of school engagement in the literature (Fredricks et al., 2004). Despite the great utility and popularity of these three subtypes, they were not proposed as a *theory* of how engagement works, or of how and why individuals become motivated to learn, such as attribution theory, social cognitive theory, intrinsic motivation and self-determination theories, expectancy-value models, goal theories, and flow theory (Schunk et al., 2014). Flow theory (Csikszentmihalyi and Csikszentmihalyi, 1988; Csikszentmihalyi, 1990, 1997) is a particularly useful and dynamic framework for characterizing the quality of engagement of an individual while engaged in an activity or task. Flow is conceptualized as a heightened state of engagement characterized by the following phenomenological aspects: (a) a merging of action and awareness (i.e., all attention is on relevant stimuli), (b) intense concentration and absorption, (c) the perception of being in control, d) loss of self-consciousness, and (e) transformation of time, i.e., typically, time seems to fly (Csikszentmihalyi, 1990; Strati et al., 2012). The chief causal mechanism of flow experiences, according to the theory, is that the challenge of the activity and skill level of the individual engaged in it are relatively high and balanced. Otherwise, the following psychological states may arise: (a) apathy, resulting from low challenge and low skill; (b) relaxation, resulting from high skill but low challenge, (c) anxiety, resutling from high challenge but low skill. Other conditions frequently giving rise to flow include (a) the activity is autotelic (i.e., a goal in and of itself), (b) goals are clear, and (c) feedback as to obtaining those goals are clear and relatively immediate (Csikszentmihalyi, 1990; Strati et al., 2012). Flow theory has been tested and supported as a model of emergent motivation and engagement in classroom contexts (Shernoff et al., 2003), and has been found to be related to the demonstration of competencies, talent development, and school performance (Nakamura, 1988; Csikszentmihalyi et al., 1993).

The dominant research methodology used to study flow has been the Experience Sampling Method (ESM; Hektner et al., 2007; Zirkel et al., 2015). In studies utilizing the ESM, study participants are signaled at intermittent times and asked to complete a record of experience (RoE) with 20–30 experiential items soliciting the respondents' perceptions of the activity as well as cognitive and emotional states. In the development of the ESM, all such items were included in the RoE because they were believed to be facets of flow (Csikszentmihalyi and Larson, 1987; Csikszentmihalyi and Rathunde, 1993). For the sake of data reduction, however, most ESM studies examined the experiential dimensions or factors emerging from these items (i.e., “the inner landscape”), and their relationships to other factors in the participants' environment (i.e., “the outer landscape”) (Csikszentmihalyi and Larson, 1984; Hektner et al., 2007). Over time, some of the experiential dimensions were utilized by researchers to represent the flow construct (most commonly, the combination of challenge and skill; e.g., Moneta and Csikszentmihalyi, 1996). The relationship between this construct and other dimensions of positive experience such as positive affect, intrinsic motivation, and self-esteem were considered empirical validation of flow theory (Csikszentmihalyi and Csikszentmihalyi, 1988; Moneta and Csikszentmihalyi, 1996). Thus, although flow was conceptualized as a unifying experience, and the ESM was originally devised as a scale of flow, the entire RoE scale was rarely used as a measure of flow. This was most likely due to a methodological artifact: the high number of items (i.e., 20–30) on the RoE, and the prevailing use of factor analysis providing evidence of multiple factors or components. Although theoretically it made sense to regard the RoE as a single scale, the possibility that all items composed a single general factor, i.e., flow, was rarely tested or utilized in measurement models.

Classroom research suggests that student engagement is a dynamic, unfolding process best understood and operationalized through repeated measures of individuals as classroom activities and contexts change (Litmanen et al., 2012; Shernoff, 2013; Salmela-Aro et al., 2016). As is the case with the present study, this conceptualization has prompted researchers interested in student engagement in classrooms to use the ESM. To do so, researchers have adapted the RoE to include more items consistent with student engagement in academic settings, and remove items that do not apply specifically to student engagement (e.g., Shernoff D. et al., 2016; Shernoff D. J. et al., 2016). Phenomenologically, the experience of student engagement can be conceptualized as similar to flow, i.e., characterized by high concentration, an autotelic activity or intrinsic interest, and positive affect or enjoyment; (e.g., Shernoff, 2013). However, such an experience in the context of academic learning in a classroom is conceptualized and labeled as student engagement rather than flow for several reasons. Brophy's (1983) conceptualization of student engagement points to some of those considerations. First and foremost, student engagement in classrooms is intended to capture enjoyment with the learning process specifically as opposed to many other processes. Such a state is cerebral in nature, and involves less emotional arousal than other flow states (as with a pumped-up football/soccer player). Secondly, classrooms are (a) work setting, (b) compulsory or at least “required,” and (c) public settings, which is extremely different than the free-choice and recreational settings in which many flow experiences are known to occur. As a result, the baseline level of intrinsic motivation can be expected to be much lower than in many other flow experiences. Third, flow is characterized by a “heightened” or “optimal” state of experience. That is, the indicators of it described earlier (e.g., *complete* absorption and *complet*e lack of self-consciousness) are high in an absolute sense; and thus, flow is typically conceptualized as a fairly dichotomous state (one is in the heightened state of flow, or not). In contrast, motivation and engagement in classrooms is conceptualized to be fairly continuous. Relatively high engagement may be expected to lead to better performance on a test or in a course; but doing well does not *require* the “heightened” engagement or motivation of flow (Shernoff, 2010).

Student engagement is largely considered to be a metaconstruct encompassing multiple emotional, cognitive, and behavioral dispositions and related, more specific constructs (e.g., intrinsic motivation) in the school setting (Fredricks et al., 2004). Few other constructs broadly encompass multiple dimensions of student experience. There is a rich and growing literature characterizing student engagement as a property of *interaction* between students and instruction, including the salience of the learning environment (National Research Council Institute of Medicine of the National Academies, 2004; Reschly and Christenson, 2012; Shernoff, 2013; Shernoff and Bempechat, 2014; Shernoff D. et al., 2016; Shernoff D. J. et al., 2016). It is one of the few constructs characterized by the quality of classroom *experiences* and *interactions*, as affected by multiple contextual influences including students (Christenson et al., 2012). Therefore, student engagement deserves to be considered as a general factor of classroom experience that exists in addition to more specific dimensions. We define a general factor of student engagement as the quality of classroom experiences, including all of students' perceptions, emotions, and cognitions in the course of interacting with instruction (Shernoff et al., 2000). This conceptualization of student engagement is supported by the phenomenological perspective of flow theory. According to this perspective, meaningful student engagement can be conceptualized as the fusion of a large range of experiential perceptions that are typical when at work (i.e., experiences are challenging, important, demanding, goal-oriented) as well as when at play (i.e., enjoyable, interesting, relaxing, exciting, emotionally and affectively positive; Shernoff, 2013). This fusion implies that students are engaged when all of these aspects of experience are *simultaneously high*. They are engaged not only in a work-like sense (i.e., concentrating on a challenging task but not enjoying the experience), or only in a play-like sense (feeling excited but that the activity is unimportant), but rather in both senses simultaneously—as when engaged in “serious play” or playful work” (Rathunde, 1993; Csikszentmihalyi and Schneider, 2000).

Based on flow theory, this conceptualization has been qualified as students' *subjective engagement* (Shernoff, 2013). While distinct from purely cognitive, affective, or behavioral subtypes of student engagement (Fredricks et al., 2004), the subjective experience of engagement generally includes affective and cognitive aspects in the course of classroom behavior. It encompasses academic emotions described by Pekrun et al. (2002) and Pekrun and Linnenbrink-Garcia (2012), but is not limited to them because emotions are only one aspect of subjective descriptions of experience from a phenomenological perspective (Husserl and Gibson, 1931; Husserl, 1980). Indeed, a great deal of the research increasingly suggests that emotions are in some sense inseparable from achievement-related thoughts and cognitions (see Pekrun and Linnenbrink-Garcia, 2012 for a review). Those interested in studying academic emotions specifically may wisely separate them from motivation- or achievement-related constructs serving as independent or dependent variables in relation to them. We also separate variables hypothesized as precursors or outcomes of student engagement, but in our case, academic emotions are included in a larger constellation of dimensions encompassed by the student engagement construct.

Engagement and flow have both been conceptualized as time- and context-varying states as well as person-varying traits or dispositions (Hektner, 1996; Csikszentmihalyi and Schneider, 2000; Christenson et al., 2012; Shernoff, 2013). Some have distinguished between “small e” engagement at a particular point in time, capital “E” engagement characterizing a sustained investment or commitment to a domain (Shernoff, 2013). Similar to Hidi and Renninger's (2006) model of interest development, a pattern of accumulating small “e” engagement can manifest in the development of capital “E” engagement. Likewise, Csikszentmihalyi and colleagues conceptualized a pattern of experiencing flow as an essential force in healthy human development as well as the evolution of the human species (Csikszentmihalyi and Larson, 1984; Csikszentmihalyi, 1993; Csikszentmihalyi and Rathunde, 1998). A person-level propensity to have frequent flow experiences has been described in terms of “the autotelic personality” (Hektner, 1996) and psychological complexity believed to result in the successful unfolding of personal potentialities (Csikszentmihalyi, 1993; Csikszentmihalyi and Rathunde, 1998). The ESM allows for variability in engagement-related emotions and perceptions to be captured in context and subsequently modeled at within-person (i.e., variation of one student's engagement throughout the class or course) and between-person (aggregated by student across time points) level of analysis (Bieg et al., 2013; Goetz et al., 2016). In keeping with these dynamic conceptualizations of engagement and flow, we utilized multilevel models to analyze ESM data allowing us to model intraindividual variability in engagement (i.e., engagement as a state), and also reliably estimate between-person variability (i.e., engagement as a trait). Although there are studies using multilevel bifactor structural equation modeling (ML-BFSEM; e.g., Scherer and Gustafsson, 2015), to our knowledge, no ESM study has yet explored a bifactor model of classroom experience, nor in a multilevel context.

In university classes occurring in large lecture halls such as those examined in the current study, the student is frequently seen as being so passive as to suggest lack of an active role in the learning process. While it is true that behavioral choices may be limited, there are several classroom practices and perceptions that can shape a students' engagement with course learning. We examined a small number of these practices, selected among the few behavioral and perceptual choices that students make in such contexts. These included: (a) seat location, (b) learning strategies such as note taking and active listening, (c) working on problems perceived as solvable as well as those students do not know how to solve, and (d) perceptions that learning activities will be evaluated. Both research on these classroom practices as well as theoretical beliefs about student engagement and flow suggested how these factors were expected to be related to engagement, as described below.

The literature is suggestive of an effect of *seat location* in university lecture courses on engagement, attitudes, and participation (Montello, 1988). Possible reasons include the belief that sitting in the back of a classroom is associated with an inferior capacity to see and hear the instructor, greater distractions, and less participatory behavior (Meeks et al., 2013). A compromised opportunity to participate in instruction and increased exposure to distractions associated with sitting in the back of the classroom would be expected to result in lower subjective involvement and concentrated attention characterizing flow and student engagement. This, in turn, would be expected to impede classroom learning and course performance. Kaplan and Talbot (1983) suggested that in environments such as large lecture halls, attention is provoked by environmental contexts and cues that capture involuntary attention, and is maintained by triggers to recover from involuntary attention fatigue such as forces of fascination. Research suggests that such cues are more forthcoming in front seats than in back seats, and that those sitting in back seats may suffer from the inability to control attention, which is considered to be a hallmark of student engagement conceptualized from the perspective of flow theory (Csikszentmihalyi, 1990; Shernoff, 2013). To the extent that there are effects of seating, the preponderance of the evidence, suggests that seats in the front and center of the classroom facilitate positive engagement and better performance relative to those in the back of the classroom.

One of the most common *learning strategies* during lecture recitation is *note taking* and attentive *listening*. However, students vary with respect to frequency of note taking, and each student's note taking can vary throughout the course. Several studies indicate that note taking is helpful for retention, recall and synthesis of material, although these capacities are enhanced by also reviewing the notes (Fisher and Harris, 1973; Carter and Van Matre, 1975; Kiewra, 1989; Kiewra et al., 1991). Few studies have examined the relationship between note taking and student engagement and experience, however; and we are not aware of any studies examining the relationship between attentive listening and engagement in university courses. Based on the research to date, we would expect that both note taking and listening would increase student engagement and flow. With respect to engagement, both strategies would be considered interactive with greater engagement, especially behavioral engagement (i.e., on-task behavior). Exercising opportunities for action is also considered a key condition enabling flow (Csikszentmihalyi, 1990).

Students in the present study were asked to indicate if they were working on *problems that are solvable*. Since solvable problems are reachable with reasonable effort, working on such problems are be expected to lead to high levels of engagement. This hypothesis is supported by flow theory, since individuals are predicted to be in flow when challenges and skills are high and in balance, as when working on a challenging but solvable problem. Students were also asked to indicate when they are working on *problems that they do not know how to solve*. Since the difficulty of these problems may overmatch students skills, this may result in anxiety according to flow theory, and thus lower levels of engagement. Nevertheless, even working on difficult problems may be more engaging than when not working on any problem, since the absence of a challenge and use of skills would be expected to result in apathy, which describes a very low level of engagement.

Research suggests that *student's expectations for evaluation* can influence students' engagement and performance in the course (Wiggins, 1993; American Psychological Association, 1997). The clear goal of obtaining skills and competencies in expectation of a performance or demonstration of competencies is associated with achievement motivation (Bempechat and Mirny, 2005) and flow (Csikszentmihalyi, 1990). Therefore, the expectation of evaluation was hypothesized to increase student engagement. In a lecture class, this expectation can be elevated during an in-class quiz, or in review classes prior to the tests.

Additionally, student engagement has been shown to function as a pathway leading to valued educational outcomes such as learning and achievement (Ladd and Dinella, 2009). For example, the extent of students' concentration, enjoyment, and interest in learning activities has been shown to predict learning and achievement outcomes (Reeve, 2013). An increasing number of studies, including large surveys of student engagement in U.S. colleges and universities such as the National Survey of Student Engagement (2016), have related active engagement in classrooms to a higher quality of student learning and higher order thinking skills (also see McKeachie, 1990), as well as to academic achievement (National Research Council Institute of Medicine of the National Academies, 2004; Kelly, 2008; See also Shernoff and Schmidt, 2008). We therefore expected for student engagement to be related to perceived learning and course performance in this study. In addition, we anticipated that student engagement would mediate the relationship between classsroom practices and student learning and performance outcomes. Student engagement has received increasing attention both because it is considered to be potentially influenced by the learning environment (i.e., teacher controllable), and because of its observed or assumed influence on learning (Willms, 2003; Fredricks et al., 2004). Although student engagement is presumed to be a mediator of the impact of the learning environment on student learning and performance, this mediating relationship is seldom tested explicitly.

Overall, we had two core aims for the study. First, we tested the appropriateness and utility of a bifactor model to characterize a sample of academic experiences from 407 undergraduate students at three time points throughout a course in financial accounting. Specifically, we sought evidence for whether or not a bifactor measurement model including a general and specific factors of classroom experience fit our data better than traditional factorial model composed only of specific factors. Second, we tested the extent to which these factors were predicted by student practices and perceptions (i.e., choice of seat location, note taking, problem solving, and expectation of evaluation), as well as the extent to which they predicted perceived learning and course performance. Because of the importance of active learning strategies and behaviors for student engagement, and the link between student engagement and performance discussed above, we expected that engagement factors would be predicted by these student-driven classroom variables, and would predict both outcomes tested.

## Methods

### Participants

Participants were students (*N* = 407) in an undergraduate Financial Accounting at the business school of a large East Coast university in the U.S. Participants were drawn from two cohorts who took the course in the fall of 2014 (*n* = 162) and fall of 2015 (*n* = 245). The course was taught by the same experienced, white, male professor at the business school in both years. All participants volunteered to participate following an informed consent procedure. A subsample of students (*n* = 339) completed the ESM. A subsample of these students (*n* = 258) also completed the background survey. Among these students, fifty percent were female; 40% were Asian, 24% were White, 20% were Hispanic or Latino, 10% were Black or African American, 4% were self-identified as “Other–Indian,” and 2% were another ethnicity; approximately 23% were not native English speakers; 82% were freshmen, 13% were sophomores, and 5% were juniors; approximately 67% were accounting, finance, or other business majors, and 33% were other majors.

A comparison of sample characteristics by cohort is presented in Table 1. All person-level variables available were compared between the two cohorts. The comparison revealed no significant differences in the composition of the two cohort subsamples with respect to gender, ethnicity, native English speakers, and college major, with the one exception of the percentage of the sample that was Latino/Hispanic. Fourteen percent of the sample was Latino/Hispanic in the Cohort 1, compared to 28% in Cohort 2. There were no significant differences with respect to prior academic achievement as measured by self-reported cumulative GPA. There were also no significant differences in the only person-level outcome, final grade in the course. In addition, the instructor and course content were identical in both years. The process for data collection was also identical. Pending an invariance analysis of classroom experience measures, the two subsamples were deemed sufficiently comparable to consider pooling in subsequent analyses in order to maximize sample size.

**Table 1:** Comparison of cohort characteristics.

	**Cohort 1**	**Cohort 2**	***T*-value**
% Female	46	48	0.24
% African American	7	10	0.08
% Asian	38	27	1.87
% Indian	6	2	1.73
% Latino/Hispanic	14	28	2.55^*^
% White	27	28	0.66
% English 1st Language	80	78	0.41
% Freshman	81	86	1.15
% Sophomore	15	10	1.42
% Junior	4	3	0.09
% Accounting, finance or other business major	72	69	0.47
Prior Cumulative GPA	2.68	2.95	1.45
Final Course Grade	2.61	2.66	0.34

*Comparison reflects only students completing the background survey (n = 258). A slightly larger group contributed ESM data*.

### Procedures

#### Background survey

A Student Background Survey was administered to all participants in one of the first classes of the fall semester in both data collection years.

#### Experience sampling method (ESM)

In both years of the course, the ESM was administered in three separate classes during the semester, roughly equally spaced apart. Classes were 80 min long. A text message was sent to the personal smartphones of all participants once in the first half, and once in the second half, of each class sampled. The instructor asked all participants to place their phones on vibrate mode. The text message provided a link to a Record of Experience (RoE) survey prepared on Qualtrics. Students then completed the RoE in about 4–5 min. In the RoE, participants reported the time of completion and 30 multiple choice and scaled items about their classroom practices, perceptions, engagement, emotions, and other aspects of their subjective experience in the moment just before receiving the text message. Students were divided into 10 groups. In the first and last half of the class, a text message was sent to each group in staggered succession within a 30 min period (approximately one group every 3 min). Therefore, each partcipant had the opportunity to complete the RoE twice in each of the three classes in which the ESM was administered (*Max* = 6). Total N_RoE_ = 1,081.

### Measures

#### Measures from background survey

For each participant, the Student Background Survey solicited gender, race/ethnicity, native language, year in which university coursework began, major subject, and cumulative GPA.

#### Experience sampling variables

Thirty items comprised the RoE. Seven items solicited students' practices and perceptions of the activity in which they were engaged at the time of the text message. Twenty-three items measured students' emotional and cognitive states on 5-point Likert-type response scales ranging from not at all to very much. Intraclass correlations (ICCs) calculated at the student level were 0.35–0.60. Additional descriptive statistics for RoE variables are provided in Table 2.

**Table 2:** Student experience and student outcome variable descriptives.

**RoE variables**	**RoE item**	***n***	***M***	***SD***	**Min**	**Max**	**ICC**
**CLASSROOM EXPERIENCE VARIABLES**							
Interest	Was it interesting?	1,078	3.13	1.11	1	5	0.56
Enjoyment	Did you enjoy what you were doing?	1,078	2.98	1.18	1	5	0.58
Excitement	Were you excited about what you were doing or learning?	1,067	2.79	1.19	1	5	0.59
Challenge	Was it challenging?	1,078	3.05	1.09	1	5	0.40
Skill	Were you using a high level of skill?	1075	2.88	1.13	1	5	0.45
Concentration	How hard were you concentrating?	1,078	3.33	1.12	1	5	0.44
Effort	How hard were you trying?	1,077	3.31	1.10	1	5	0.52
Importance	How important was this activity or topic to you?	1,079	3.79	1.05	1	5	0.55
Goal Clarity	Were the goals clear?	1,077	3.85	0.97	1	5	0.48
Relevance	Do you believe that the topic or activity was or will be relevant, useful or practical for you current or future goals or jobs?	1,077	3.94	1.20	1	5	0.62
Effective Instruction	How much did what you were doing in the class help you with your learning or understanding?	1,072	3.51	1.12	1	5	0.56
Control	Did you feel in control?	1,077	3.05	1.17	1	5	0.51
Belongingness	Did you feel important or needed?	1,077	2.79	1.34	1	5	0.57
Participation	Were you participating or asking questions?	1,070	2.42	1.27	1	5	0.39
Good Mood	How good or positive was your mood (i.e., feeling happy and vibrant)?	1,067	3.16	1.20	1	5	0.46
Successful	Did you feel successful or that you could succeed?	1,064	3.45	1.16	1	5	0.52
Detachment	Do you wish you were doing something else?	1,076	3.08	1.35	1	5	0.60
Mind Wandering	Was your mind wandering?	1,072	2.75	1.19	1	5	0.47
Boredom	Did you feel bored?	1,066	2.66	1.26	1	5	0.55
Irritation	Did you feel irritated or upset?	1,066	1.93	1.17	1	5	0.42
Learning Interference	Was something interfering with your learning?	1,062	2.24	1.20	1	5	0.42
Attention	What was the main thing you were thinking about?—The work or subject matter of this class.	1,078	.68	.47	0	1	0.35
**Student outcomes**	**Description**	***n***	***M***	***SD***	**Min**	**Max**	**ICC**
Perceived Learning	How much were you learning?	1,073	3.69	1.07	1	5	0.59
Course grade	Final recorded grade in course.	290	2.63	1.09	0	4	

##### Classroom practices and perceptions

Four classroom practices and perception variables were considered as independent variables—seat location; learning strategies (e.g., note taking, effortful listening), perception of in-class problems, and expectations for evaluation:

- *Seat location*. Students' seat location was captured by the item, “Where were you sitting when you were texted?” Responses options were: (a) Back of the room, (b) Middle of the room, and (c) Front of the room.
- *Classroom learning strategies*. The RoE included the item, “What was the main thing you were doing?” Response categories were “*taking notes*,” “*trying to listen to the instructor*?,” and “Other.”
- *Working on in-class problems*. Students were also asked to indicate whether they were “working on a problem that I knew how to solve,” “working on a problem that I didn't understand,” or “other” (i.e., not working on a problem).
- *Expectation for evaluation* was indicated by the item, “Were you working on something that is going to be graded? Response categories were (a) Yes and (b) No.

##### Student engagement and experience variables

At total of 22 items on the RoE were considered as student engagement variables. See Table 2.

#### Student outcome variables

Two student outcome variables were considered—perceived learning and course performance:

- *Perceived Learning*. Perceived learning was measured by the item, “How much were you learning?” (See Table 2).
- *Course performance*. Course performance was operationalized as students' final course grade, which was provided by the instructor.

### Analytic approach

The first goal of the study was to test a two-level bifactor model compared to an approach based on traditional factor analysis, frequently performed at only one level. In the bifactor analytic approach, a general factor is expected to represent the commonality among items measuring a construct. The RoE measured the multifaceted construct of student engagement. We propose a general experiential factor of student engagement that is composed of the 21 RoE items that measure subjective experience in the moment (the 22nd item, attention, was conceptualized as a single-item variable and was not considered as a part of the factorial structure of the measurement model). In a bifactor measurement model, the first factor is a general factor consisting of all items on the scale. The remaining factors are specific factors explaining only the residual variation that the general factor does not explain. Each item of a measurement model using latent variables is typically included in the general latent variable and one of the specific latent variable representing the factor to which it loads most highly after loading onto the specific factor. A unique property of a bifactor model is that it constrains the relationship between the general and specific factors, and among the specific factors, to be uncorrelated. This eliminates the multicollinearity that would otherwise threaten the validity of the results. The uncorrelated specific factors represent unique dimensions that are not accounted for by the shared variance of all items in the general factor, and when used in subsequent predictive modeling, provide a high degree of clarity on how different constructs uniquely predict outcomes.

We utilized two-level exploratory factor analyses (EFA) in MPlus 7.11 (Muthén and Muthén, 1998/2012) to generate factor structures. In the two level-models, repeated records of experience (RoEs, level one) were nested within students (level two). The best fitting two-level bifactor model produced by EFA in MPlus using the bi-geomin rotation informed the factorial structure examined in confirmatory factor analysis (CFA); and the best fitting two-level traditional (i.e., non-bifactor) models were suggested with EFA using the geomin rotation. In both cases, latent variables were created from significantly loading items in the EFAs, and the factorial models were examined using CFA. Although only items with significant loadings were retained in latent variables representing factors, one exception was the general factor of the bifactor model, which includes all of the items. The negative residual variances of one item (effective instruction at level two) was addressed by constraining its variance to zero. Model fit from the CFAs of a two-level bifactor model was compared to the two best fitting two-level traditional models. We expected that measurement models could be different at each level, as has been shown elsewhere (e.g., Schweig, 2014). The best fitting model was utilized as the measurement model for predictive analyses.

With respect to model fit statistics, the null model (i.e., no associations specified between variables) had a root mean square error of approximation (RMSEA) that was very low (<0.012), and in such cases incremental fit indices such as the comparative fit index (CFI) and Tucker-Lewis Index (TLI) are limited to less than the typical acceptable cutoff value of 0.90 (Wallace et al., 2016). We therefore report the CFI for all CFA models, but we do not interpret it in evaluating overall model fit. Regarding other indices of model fit, and in line with recommendations for single level models (Hu and Bentler, 1999), we consider an RMSEA equal to or lower than 0.06 and 0.08, respectively, to indicate adequate to excellent fit. A proposed cutoff value of 0.08 or lower is suggested for the standardized root-mean-square residual (SRMR), which is a level-specific absolute fit index (Hu and Bentler, 1999; Wagner et al., 2013). Akaike and Bayesian Information Criteria (AIC and BIC, respectively), were also utilized for purposes of comparing model fit, with lower values indicating better fit.

In addition to model fit, we also examined the correlations among the latent variables in the traditional CFA models. High correlations among latent variables can impair the validity of results in predictive modeling due to multicollinearity.

To determine whether engagement and related experiential factors were measured similarly between the Cohort 1 and Cohort 2 subsamples, we ran a series of invariance tests on the individual level (between student) measurement model. We created aggregate individual level variables by taking the mean of all time-specific responses for each student. We then conducted a multi-group CFA on the individual level data, first estimating a configural model in which all factor loadings and item intercepts were freely estimated across the two student groups. Following Cheung and Rensvold (2002) recommendations for determining whether more constrained invariance models fit as well as less constrained models, we compared the CFIs of the configural model to a metric invariance model in which we constrained the factor loadings to be equal across groups. The metric model was then compared to a scalar invariance model that further constrained the item intercepts to be equal across groups. As suggested by Cheung and Rensvold (2002), we interpreted a change in CFI of less than 0.01 between the configural and the metric model, and similarly between the metric and scalar invariance models, as evidence that the latent engagement factors were measured similarly in the two groups. We also examined mean differences in the factors in the scalar invariance model.

The second goal of the study was to determine the degree to which classroom practices and perceptions were associated with engagement-related factors, and the extent to which engagement factors were associated with perceived learning and course grades, conceptualized as outcomes. This aim was pursued by constructing two multilevel structural equation models (MSEM) predicting each of the outcomes. Latent variables representing student engagement factors were regressed on classroom practices and perceptions; and student outcomes were regressed on latent variables representing engagement factors as well as student practices and perceptions. The final models included only statistically significant relationships after controlling for student-level covariates including cohort; gender; race/ethnicity; English as one's native language; year of study participation; and business, finance, or related major. Models would not converge with student classroom practice and perception variables (i.e., working on good problems and hard problems, listening, taking notes, and expectation of evaluation) allowed to vary between students (i.e., at level two), except for the seating location variables. Further inspection revealed unusually high standard errors of the unstandardized regression coefficients at level two but not level one for these variables, indicating that these estimates were reliable at level one, but not at level two. Therefore, they were excluded from the final level-2 models. Finally, we tested for mediation where indirect paths through engagement variables were statistically significant. Confidence intervals for the indirect estimates were estimated using a Monte Carlo procedure (MacKinnon et al., 2004).

At level two, 100% of the analytic sample completed the ESM, and 258 of 339 participants (76%) contributed the Background Survey information. At level one, approximately 3% of the RoE data was missing. Approximately 70% of level one observations had complete data on all variables, and 27% had missing data on Background Survey variables including ethnicity, gender, English as a first language, and major. To account for missing data, full-information maximum likelihood (FIML) in Mplus 7.11 was utilized for estimations using the MLR estimator (maximum likelihood estimation with robust standard errors, Muthén and Muthén, 1998/2012). In FIML, parameter estimates and standard errors for the missing values are computed with all available information in the data (Buhi et al., 2008).

## Results

### Measurement model comparisons and selections

The first goal of the study was to examine the degree to which a two-level bifactor model, with student engagement proposed as a general factor of students' classroom experience, was an appropriate alternative to a traditional two-level factorial model with no general factor.

#### Factorial structures and model comparisons

Preliminary bifactor and non-bifactor factorial structures were suggested by two-level exploratory factor analyses (EFA) using the bi-geomin rotation (for bifactor models) and geomin rotation (for traditional models) in MPLUS 7.11. Bifactor models with between two (one general and one specific) and six factors were considered, with a two factor solution the minimum by definition. In general, models with more factors offered better model fit, although the solution with six factors at both levels did not converge. Best fit was offered by the model with five within-student factors and six between-student factors, RMSEA = 0.03, SRMR (Within) = 0.03, SRMR (Between) = 0.02, CFI = 0.97, TLI = 0.94; and the model with five within-student factors and five between-student factors, RMSEA = 0.03, SRMR (Within) = 0.03, SRMR (Between) = 0.03, CFI = 0.96, TLI = 0.93). However, the model with six between-student factors included two between-level factors which had only two significantly loading items including one low-loading item (i.e., < 0.32). Therefore, a solution with five within-student factors and five between-student factors was preferred.

We here describe results of factor loadings of the exploratory factor analysis (EFA) for this model; factors and loadings of the same model in the context of confirmatory factor analysis (CFA) are presented in Table 3. All of the 21 items significantly loaded onto the general factor except for one (challenge) at level one, and except for challenge and learning interference at level two. All items loaded positively onto the general factor at both levels except for detachment, irritation, boredom, mind wandering, and learning interference, which contributed negative loadings. At level one, all the items except for two (mind wandering and effective instruction) also loaded significantly onto at least one other specific factor of classroom experience. The highest of these loadings determined the formation of specific factors. Specific factors were labeled according to an interpretation of the composite of high loading items as a within-person, time varying variable. Those factors were: Intrinsic Motivation (three items: enjoyment, interest, and excitement), Academic Intensity (eight items: challenge, skill, effort, concentration, detachment, irritation, boredom, and learning interference), Salience: (three items: importance, relevance, and goal clarity), and Classroom Self-Esteem (five items: successful, belongingness, good mood, control, and participation). With eight items loading positively onto Academic Intensity, this factor appeared to represent a simultaneous elevation of challenge, cognitive demands, as well as an accompanying emotionality of frustration and resignation. It is important to remember, however, that the specific factors explain variation after the formation of the general factor, student engagement, onto which challenge, skill, concentration, and effort loaded positively (with the loading for challenge close to zero), and detachment, irritation, boredom, and learning interference loaded negatively.

**Table 3:** Standardized and unstandardized factor loadings and model fit: two-level, bifactor CFA.

**Level-one variables**	**Student engagement β (B)**	**Intrinsic motivation β (B)**	**Academic intensity β (B)**	**Salience β (B)**	**Classroom self-esteem β (B)**
Interest	0.55 (0.40)	0.43 (0.32)			
Enjoyment	0.51 (0.39)	0.61 (0.47)			
Excitement	0.39 (0.29)	0.33 (0.25)			
Challenge	0.09 (0.08)^a^		0.48 (0.41)		
Skill	0.47 (0.39)		0.42 (0.35)		
Concentration	0.53 (0.45)		0.25 (0.21)		
Effort	0.53 (0.40)		0.43 (0.33)		
Detachment	−0.33 (−0.28)		0.37 (0.32)		
Irritation	−0.22 (−0.20)		0.36 (0.33)		
Boredom	−0.43 (−0.36)		0.28 (0.24)		
Lrn. Interfere	−0.21 (−0.20)		0.22 (0.20)		
Importance	0.49 (0.34)			0.45 (0.32)	
Goal Clarity	0.42 (0.29)			0.29 (0.20)	
Relevance	0.25 (0.19)			0.43 (0.32)	
Control	0.43 (0.35)				0.28 (0.22)
Belongingness	0.26 (0.23)				0.38 (0.33)
Participation	0.19 (0.20)				0.25 (0.25)
Good Mood	0.40 (0.35)				0.29 (0.25)
Successful	0.35 (0.28)				0.42 (0.33)
Mind Wander	−0.45 (−0.40)				
Effective Inst.	0.50 (0.37)				
**Level-two variables**	**Student engagement β (B)**	**Work orientation β (B)**	**Learning orientation β (B)**	**Classroom self-esteem β (B)**	**Disengagement β (B)**
Interest	0.96 (0.80)	−0.20 (−0.16)			
Enjoyment	0.95 (0.87)	−0.25 (−0.23)			
Skill	0.79 (0.57)	0.43 (0.31)			
Concentration	0.80 (0.56)	0.51 (0.36)			
Effort	0.70 (0.53)	0.63 (0.48)			
Importance	0.80 (0.61)		0.28 (0.21)		
Goal Clarity	0.60 (0.39)		0.57 (0.38)		
Relevance	0.66 (0.63)		0.49 (0.46)		
Effective Inst.	0.82 (0.68)		0.52 (0.42)		
Control	0.69 (0.57)			0.31 (0.26)	
Good Mood	0.74 (0.60)			0.21 (0.17)	
Successful	0.79 (0.65)			0.55 (0.46)	
Challenge	0.10 (0.07)^a^				0.45 (0.29)
Detachment	−0.68 (−0.71)				0.55 (0.58)
Mind Wander	−0.57 (−0.46)				0.67 (0.54)
Irritation	−0.30 (−0.22)				0.73 (0.54)
Boredom	−0.74 (−0.69)				0.61 (0.57)
Ln. Interfere	−0.08 (−0.06)^a^				0.75 (0.58)
Excitement	0.96 (0.88)				
Belongingness	0.75 (0.75)				
Participation	0.47 (0.38)				
**MODEL FIT**					
RMSEA	0.044				
SRMR (Within)	0.053				
SRMR (Between)	0.080				
CFI	0.881				
Akaike (AIC)	57,898.08				
Bayesian (BIC)	58,606.04				

*CFA, Confirmatory factor analysis; Ln. Interfere, Learning Interference; CFI, comparative fit index; RMSEA, root mean error of approximation; SRMR, standardized root mean square residual. AIC, Akaike Information Criteria. BIC, Bayesian Information Criteria*.

a *Coefficient not significant*.

At level two, all items loaded significantly onto a specific factor except for excitement, belongingness, and participation. Four level-two specific factors emerged, labeled according to the interpretation of the composite of items considered as a variable that varies across students: Work Orientation (five items: effort, concentration, skill, interest—negative loading, and enjoyment—negative loading), Learning Orientation (four items: goal clarity, effective instruction, relevance, and importance), Classroom-Self Esteem (three items: successful, control, and good mood), and Disengagement (six items: learning interference, irritation, mind wandering, boredom, detachment, and challenge). Similarly to the level-one Academic Intensity factor, the level-two Work Orientation factor involved concerted effort and a negative correlation to positive emotions, whereas those same positive emotions loaded positively onto the level-two general (engagement) factor.

Factorial structures for two-level traditional (non-bifactor) models were informed by EFA with geomin rotation. Solutions with between 1 and 5 factors at both levels were considered. The best fitting models were composed of (a) five within-student factors and five between-student factors, and (b) five within-student factors and four between-person factors. Model fit for both models was very good. For the 5-within and 5-between factor model, RMSEA = 0.03, SRMR (Within) = 0.03, SRMR (Between) = 0.03, CFI = 0.96, TLI = 0.93; for the 5-within and 4-between factor model, RMSEA = 0.04, SRMR (Within) = 0.03, SRMR (Between) = 0.04, CFI = 0.95, TLI = 0.92.

We here describe factor loadings of the EFA for the traditional models; CFA factors and loadings of the same models are presented in Table 4. All items loaded significantly onto at least one factor in both models. For both models, the within-student 5-factor structure was identical: Intrinsic Motivation (three items: enjoyment, interest, and excitement), Flow (four items: challenge, skill, concentration, and effort), Salience (four items: importance, relevance, goal clarity, and effective instruction), Classroom Self-Esteem (five items: successful, control, good mood, belongingness, and participation), and Disengagement (five items: boredom, detachment, mind wandering, irritation, and learning interference). At level two, the five-factor solution produced the following factors: Intrinsic Motivation (five items: enjoyment, interest, excitement, importance, and detachment—negative loading), Flow/Autotelic Personality (five items: challenge, skill, concentration, effort, and participation), Learning Orientation (three items: effective instruction, goal clarity, and relevance), Classroom Self-Esteem (four items: successful, good mood, control, and belongingness), and Disengagement (four items: boredom, mind wandering, irritation, and learning interference). The factors were the same for the between-student four factor solution, except that there was no Classroom Self–Esteem factor. Instead, the control and belongingness items loaded onto the Intrinsic Motivation factor, and control, successful, and importance loaded onto the Learning Orientation factor.

**Table 4:** Standardized and unstandardized factor loadings and model fit: two-level, traditional (non-bifactor) CFAs.

**Level-one variables**		**Intrinsic motivation β (B)**	**Flow β (B)**	**Salience β (B)**	**Classroom self-esteem β (B)**	**Disengagement β (B)**
Interest		0.74 (0.56)				
Enjoyment		0.76 (0.59)				
Excitement		0.53 (0.41)				
Challenge			0.27 (0.23)			
Skill			0.61 (0.50)			
Concentration			0.58 (0.47)			
Effort			0.65 (0.50)			
Importance				0.65 (0.45)		
Goal Clarity				0.51 (0.35)		
Relevance				0.43 (0.32)		
Effective Instruction				0.47 (0.34)		
Control					0.55 (0.44)	
Belongingness					0.42 (0.37)	
Participation					0.31 (0.31)	
Good Mood					0.49 (0.43)	
Successful					0.55 (0.44)	
Detachment						0.52 (0.45)
Mind Wandering						0.52 (0.45)
Irritation						0.34 (0.30)
Boredom						0.62 (0.53)
Learning Interference						0.24 (0.22)
**Level-two variables**		**Intrinsic motivation β (B)**	**Flow/autotelic β (B)**	**Learning orientation β (B)**	**Classroom self-esteem β (B)**	**Disengagement β (B)**
**5 LEVEL-TWO FACTORS**						
Interest		0.98 (0.80)				
Enjoyment		0.97 (0.88)				
Excitement		0.94 (0.85)				
Challenge			0.25 (0.17)			
Skill			0.91 (0.67)			
Concentration			0.96 (0.71)			
Effort			0.91 (0.71)			
Importance		0.81 (0.62)				
Goal Clarity				0.80 (0.53)		
Relevance				0.76 (0.71)		
Effective Instruction				1.00 (0.83)		
Control					0.76 (0.64)	
Belongingness					0.73 (0.72)	
Participation			0.52 (0.41)			
Good Mood					0.79 (0.65)	
Successful					0.88 (0.73)	
Detachment		−0.80 (−0.80)				
Mind Wandering						0.80 (0.65)
Irritation						0.67 (0.50)
Boredom						1.00 (0.91)
Learning Interference						0.51 (0.40)
**MODEL FIT**						
RMSEA	0.050					
SRMR (Within)	0.061					
SRMR (Between)	0.120					
CFI	0.839					
Akaike (AIC)	58,183.11					
Bayesian (BIC)	58,796.34					
**4 LEVEL-TWO FACTORS**						
Interest		0.97 (0.78)				
Enjoyment		0.97 (0.84)				
Excitement		0.95 (0.86)				
Challenge			0.26 (0.17)			
Skill			0.90 (0.67)			
Concentration			0.96 (0.71)			
Effort			0.91 (0.71)			
Importance				0.83 (0.63)		
Goal Clarity				0.79 (0.53)		
Relevance				0.78 (0.74)		
Effective Instruction				0.95 (0.79)		
Control				0.71 (0.58)		
Belongingness		0.69 (0.68)				
Participation			0.51 (0.40)			
Good Mood		0.77 (0.62)				
Successful				0.86 (0.70)		
Detachment		−0.80 (−0.81)				
Mind Wandering						0.79 (0.64)
Irritation						0.66 (0.50)
Boredom						1.00 (0.92)
Learning Interference						0.51 (0.39)
**MODEL FIT**						
RMSEA	0.050					
SRMR (Within)	0.062					
SRMR (Between)	0.122					
CFI	0.836					
Akaike (AIC)	58,197.78					
Bayesian (BIC)	58,796.06					

*CFA, Confirmatory factor analysis; Autotelic, Autotelic Personality; CFI, comparative fit index; RMSEA, root mean error of approximation; SRMR, standardized root mean square residual. AIC, Akaike Information Criteria. BIC, Bayesian Information Criteria*.

*^a^ Coefficient not significant*.

In proceeding to confirmatory factor analysis (CFA), latent variables were constructed for each factor with the composite items. CFA factor loadings onto these latent variables and model fit of the bifactor model and for the traditional (non-bifactor) models are presented in Tables 3, 4, respectively. In response to our first study aim, results reveal that model fit of the two-level bifactor model (Table 3) was better than that for either of the traditional (non-bifactor) models (Table 4). For the bifactor model, RMSEA = 0.044, SRMR (Within) = 0.053, SRMR (Between) = 0.080, CFI = 0.881, Akaike (AIC) = 57898.08, Bayesian (BIC) = 58606.04. For the 5-factor (within) and 5-factor (between) traditional model, RMSEA = 0.050, SRMR (Within) = 0.061, SRMR (Between) = 0.120, CFI = 0.839, Akaike (AIC) = 58183.11, Bayesian (BIC) = 58796.34. For the 5-factor (within) and 4-factor (between) traditional model, RMSEA = 0.050, SRMR Within = 0.062, SRMR Between = 0.122, CFI = 0.836, Akaike (AIC) = 58197.78, Bayesian (BIC) = 58796.06.

A further advantage of the bifactor model was that the correlations between all of the latent variables, including the correlations between the general factor and specific factors and the correlations among all combinations of specific factors, are all constrained to zero by definition. While a detailed examination of the correlations among latent variables in the traditional models is not necessary to address the aims of our study, a cursory inspection reveals that these correlations were moderate to high. At level one, correlations in the traditional model ranged from 0.22 to 0.70 (specifically, *r*s = 0.22, 0.27, 0.37, 0.39, 0.54, 0.58, 0.58, 0.60, 0.64, and 0.70). At level two, the correlation among the latent variables in the traditional 5-factor model ranged from 0.58 to 0.89 (specifically, *r*s = 0.58, 0.69, 69, 0.74, 0.75, 0.77, 0.80, 0.81, 0.86, and 0.89); and in the 4-factor model at level two, they ranged from 0.57 to 0.86 (specifically, *r*s = 0.57, 0.69, 0.72, 0.77, 0.81, and 0.87). Particularly at level two, the traditional models could not be used with confidence in predictive modeling due to the potential for significant multicollinearity. The bifactor model avoids this fatal flaw. Combined with its superior fit, the bifactor model was therefore used for predictive modeling in pursuit of our second study aim.

#### Selection and examination of the bifactor measurement model

We next inspected the bifactor model in terms of the reliabilities of the latent variables, the ICC of the engagement variable, and invariance of between-student latent variables between Cohort 1 and Cohort 2.

A reliability analysis was conducted on all the composite factors composing the latent variables in the model. Cronbach's alpha was computed at level one for all within-person factors, and at level two for all between-student factors. Reliabilities of within-person factors were as follows: For Student Engagement (general factor), α = 0.91; For Intrinsic Motivation, α = 0.89; For Academic Intensity, α = 0.52; For Learning Orientation, α = 0.75; For Class Self-Esteem, α = 0.74. Reliabilities of level-two factors were: For Student Engagement (general factor), α = 0.93; For Work Orientation, α = 0.59; For Learning Orientation, α = 0.85; For Class Self-Esteem, α = 0.79; For Disengaged, α = 0.81. The within-person factor, Academic Intensity, and the between-person factor, Work Orientation, were not utilized in predictive modeling due to insufficient reliability.

With respect to the ICC of the engagement variable, an examination of the level-one and level-two variances of the Student Engagement latent variable in a fully unconditional model (i.e., no predictors) revealed that 69% of its variation resided between students, and 31% was within students.

Results of the invariance testing to determine whether engagement was being measured similarly across the two subsamples of students from the two consecutive courses were as follows. The change in CFI between the configural and metric model was below the 0.01 cutoff suggested by Cheung and Rensvold (2002). We therefore proceeded to check for scalar invariance by further constraining the item intercepts to be equal across groups. The change in CFI from the metric to scalar model was similarly less than 0.01. Passing this invariance test means that the latent variables were similar measures in the two subsamples. We also examined the mean differences in the measures between the two groups in the scalar invariance model. Mean differences in student engagement, learning orientation, and classroom self-esteem were not statistically significant. Mean Work Orientation was lower in Cohort 1 than in Cohort 2 (mean difference = −0.153, *p* < 0.05) and mean Disengagement was higher in Cohort 1 (diff = 0.452, *p* < 0.001). However, Disengagement was dropped from predictive modeling due to low reliability. Overall, evidence of scalar invariance across students in the two groups demonstrated that the between-student level bifactor model was a good fit to the data for students in both groups. Combined with the analyses showing minimal differences in sample composition, this allowed us to confidently proceed with structural analyses using the pooled sample of Cohort 1 and Cohort 2 students.

### Two-level structural models predicting perceived learning and course performance

The second aim of the study was to test the predictive relationships embedded in the models: the influence of classroom practices and perceptions on student engagement and specific factors of classroom experience; and the influence of classroom experience variables on perceived learning and final grade in the course.

**Figure 1:**
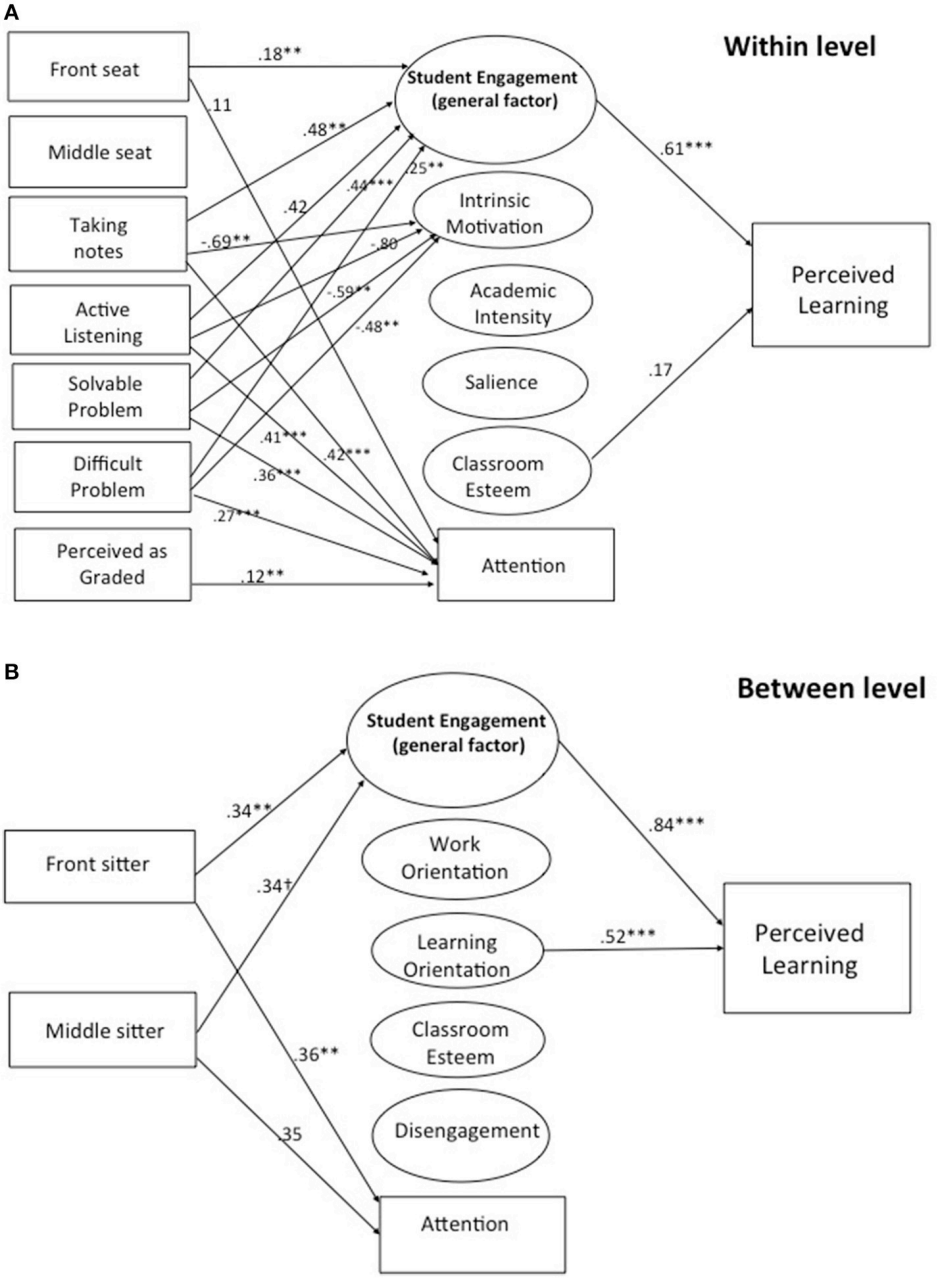
**(A)** Two-Level structural equation model with perceived learning as outcome–within-student level. Unstandardized coefficients are shown. All paths are significant at *p* < 0.05. ^**^p < 0.10 and ^***^p < 0.001. Reference category for the seating variables is sitting in the back of the classroom. Front Seat, Sitting in the front of the classroom; Middle Seat, Sitting in the middle of the classroom; Solvable Problem, Completing a solvable problem; Difficult Problem, Completing a difficult problem; Perceived as Graded, Activity perceived to be evaluated or graded; Classroom Esteem, Classroom Self-Esteem. **(B)** Two-Level structural equation model with perceived learning as outcome——between-students Level. Unstandardized coefficients are shown. All paths are significant at *p* < 0.05. ^**^p < 0.10 ^***^p < 0.001, and ^†^*p* < 0.10. Reference category for the seating variables is sitting in the back of the classroom. Additional controls at the between (student) level include gender; race/ethnicity; business, finance, or related major; English as one's native language, and year of study participation. Front Seat, Sitting in the front of the classroom; Middle Seat, Sitting in the middle of the classroom; Classroom Esteem, Classroom Self-Esteem.

**Figure 2:**
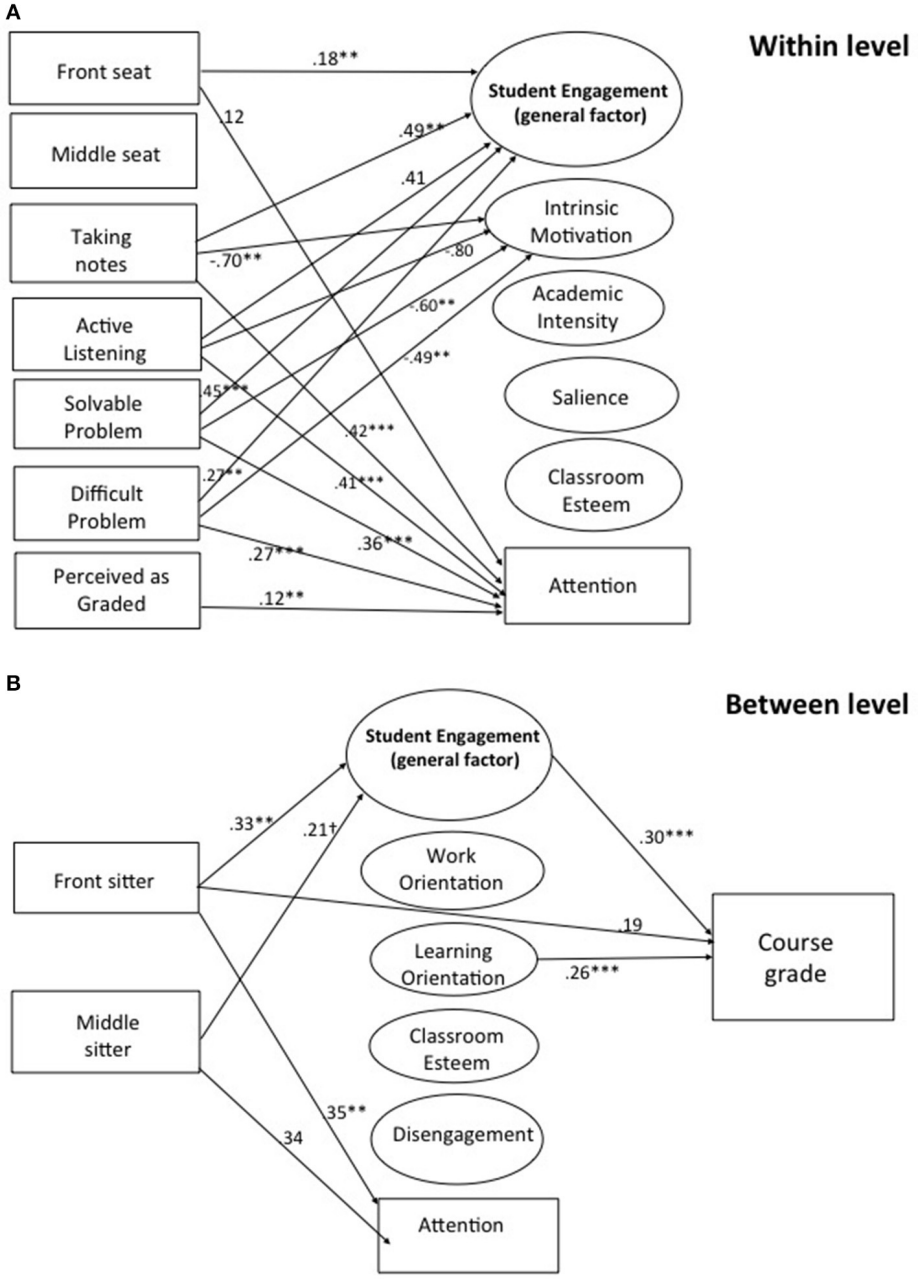
**(A)** Two-Level structural equation model with course grade as outcome—within-student level. Unstandardized coefficients are shown. All paths are significant at *p* < 0.05. ^**^p < 0.10 and ^***^p < 0.001. Reference category for the seating variables is sitting in the back of the classroom. Front Seat, Sitting in the front of the classroom; Middle Seat, Sitting in the middle of the classroom; Solvable Problem, Completing a solvable problem; Difficult Problem, Completing a difficult problem; Perceived as Graded, Activity perceived to be evaluated or graded; Classroom Esteem, Classroom Self-Esteem. **(B)** Two-Level structural equation model with course grade as outcome—between-students Level. Unstandardized coefficients are shown. All paths are significant at *p* < 0.05. ^**^p < 0.10, ^***^p < 0.001, and ^†^*p* < 0.10. Reference category for the seating variables is sitting in the back of the classroom. Additional controls at the between (student) level include gender; race/ethnicity; business, finance, or related major; English as one's native language, and year of study participation. Front Seat, Sitting in the front of the classroom; Middle Seat, Sitting in the middle of the classroom; Classroom Esteem, Classroom Self-Esteem.

Results of multilevel structural equation models (MSEM) with perceived learning as the outcome are presented in Figure 1A (level-one paths) and Figure 1B (level-two paths); and results with course grade as the outcome are presented in Figure 2A (level-one paths) and Figures 2B (level-one paths). Only statistically significant paths are shown, and standardized regression coefficients are shown. For both models, the independent variables, trying to listen, working on solvable and difficult problems, and the perception that the activity was graded were represented as within-student variables only due to lack of model convergence when including them in the level-two models. Therefore, the seating location variables were the only independent variables represented at level two. The effects of student-level background variables on the outcome (i.e., perceived learning in Figure 1 and course grades in Figure 2) were controlled.

Figure 1A illustrates that within-student variation in sitting in the front of the classroom (compared to the default category of sitting in back), taking notes, active listening, and working on both solvable and difficult problems was significantly related to within-student variation in student engagement (general factor). This means that students who sat in the front and back of the classroom at least once were, on average, more engaged when sitting in front. Similarly, students were more engaged when taking notes, listening, and solving problems than when not doing so. Student engagement, in turn, was positively related to perceived learning. We next tested mediation for all significant indirect paths. A significant mediation relationship was confirmed for the effect of sitting in the front of the class, working on good problems, working on hard problems, taking notes, and active listening on perceived learning as mediated by student engagement (for the path starting with sitting in front, indirect = 0.28 [CI = 0.09, 0.49]; for the path starting with solvable problems, indirect = 0.63, [CI = 0.30, 0.97]; for the path starting with difficult problems, indirect = 0.44 [CI = 0.10, 0.79]; for the path starting with taking notes, indirect = 0.52 [CI = 0.18, 0.86]; for the path starting with active listening, indirect = 0.37 [CI = 0.06, 0.70]. Sitting in the front of the class, taking notes, active listening, working on both solvable and difficult problems, and the perception that the activity would be evaluated also had a positive effect on attention. Taking notes, active listening, and working on solvable and difficult problems also had a negative effect on the intrinsic motivation specific factor.

Figure 1B depicts the between-student model with perceived learning as the outcome. Between-student variation in sitting in the front of the class was positively related to between-student variation in student engagement. This means that students who had the propensity to sit in the front of the class more so than other students (labeled in the figure as “front sitters”) reported higher average student engagement compared to other students. In turn, higher average student engagement positively predicted higher average perceived learning. The test of the indirect effect of the tendency to sit in front on average perceived learning as mediated by student engagement was significant, indirect = 0.50 [CI = 0.16, 0.82]. The student-level patterns both of sitting in front and middle seats compared to back seats were both associated with higher average attention in class. Finally, average learning orientation was related to average perceived learning. Model fit of the MSEM with perceived learning as outcome was as follows: RMSEA = 0.04, SRMR (Within) = 0.06, SRMR (Between) = 0.15, CFI = 0.84.

Figure 2 illustrates the multilevel structural equation models with course grade as the outcome variable. Because course grade varied only between persons, it did not enter the within-student model (Figure 2A). Statistical significance of the remaining relationships, between classroom practices/perceptions and classroom experience variables, were without exception the same as depicted in Figure 1A (discussed above). In the between-student part of the model, illustrated in Figure 2B, the tendency to sit in front seats had a direct effect on course grade. The pattern of sitting in front also had a positive effect on student engagement and attention, and the tendency to sit in middle seats (compared to sitting in back seats) also had a positive effect on attention, just as in Figure 1B. Student engagement and learning orientation both predicted course grade. The indirect effect of sitting in the front seats on course grades as mediated by average engagement was significant, indirect = 0.26 [CI = 0.09, 0.50]. Model fit statistics for the MSEM with course grade as outcome was: RMSEA = 0.04, SRMR (Within) = 0.06, SRMR (Between) = 0.15, CFI = 0.83.

## Discussion

This study provides an enriched understanding of the way in which students' classroom practices and perceptions in a university gateway course relate to the quality of student engagement and experience during class time. The study also enhances our understanding of student engagement as reflective of general vs. specific aspects of subjective experience, suggesting the methodological feasibility and utility of a bifactor approach for modeling experiential data. These insights are especially significant given the increased international attention paid to the importance of engagement as central to educational outcomes such as participation, belongingness, academic achievement, and retention versus dropout (Christenson et al., 2012; Eccles, 2016; National Survey of Student Engagement, 2016).

The first aim of the study was to test whether a conceptualization of engagement from the perspective of flow theory reflective of a general or unidimensional aspect of student experience was supported by classroom data from students taking an undergraduate course in financial accounting. Toward that end, we compared a two-level bifactor model and two traditional (non-bifactor) two-level models (one with five within-student factors and five between-student factors, and one with five within-student factors and four between-student factors). We found that the bifactor model fit the data in this study better than both of the traditional models. Moreover, there were high correlations among the latent variables in the traditional models, especially at the between-student level, where correlations ranged between 0.57 and 0.89. In the bifactor model, on the other hand, the correlations among all latent variables were constrained to be zero. Thus, this study demonstrated the utility of techniques in predictive models drawing on multiple aspects of classroom experience that mitigate issues of multicollinearity associated with those aspects. Combined with the superior fit of the bifactor model, the study provides some preliminary evidence of the advantages of utilizing a multilevel and bifactor approach in studying classroom experience, and of using it in combination with multilevel models (e.g., multilevel bifactor structural equation modeling, or ML-BFSEM). Nevertheless, this study also supported the proposition that there are other, specific aspects of student classroom experience beyond a general student engagement factor.

A second aim of the study was to examine the extent to which classroom practices and perceptions predicted student engagement and other classroom experience factors, and the extent to which engagement factors may predict student learning and course grades. The expectation that sitting nearer to the front of the class, taking notes, active listening, and working on problems would be related to student engagement was supported as a within-person effect. In addition, the perception that the activity would be evaluated had a positive effect on attention. In turn, student engagement predicted perceived learning. The indirect effect of sitting in the front of the class (compared to sitting in back), working on both solvable and difficult problems, taking notes, and active listening on perceived learning as mediated by student engagement was significant.

Between students, the tendency to sit in front seats predicted average student engagement, which in turn predicted both average perceived learning and course grades. The tendency to sit in front seats also had a positive, direct effect on grades. The indirect effect of the tendency to sit in the front and middle seats on both average perceived learning and course grades as mediated by student engagement was also significant. Learning orientation also had a positive effect on both perceived learning and grades.

### General and specific aspects of student classroom experience, perceptions, and emotions

In the preferred bifactor measurement model, all ESM experiential items loaded onto the general factor of classroom experience, conceptualized as student engagement. Student engagement reflected all positive perceptions, motivations, emotions, and cognitions measured as students interacted with classroom instruction, and inversely reflected negative emotions and experiences. The fact that a measurement model with this general student engagement factor fit the data better than those featuring only specific factors suggests that prevailing arguments regarding the multidimensional nature of engagement may be oversimplified. Overlooked is that student engagement may have a strong general element that includes perceptional, motivational, emotional, and cognitive aspects of students' classroom experience. This support of a general aspect of student engagement corroborates previous studies finding that all of the diverse ESM items on the RoE administered in high school classrooms fits the Rasch model very well, demonstrating a strong unidimensional aspect of students' classroom experience according to Rasch analysis (Cavanagh and Shernoff, 2013, 2014).

The student engagement factor reflects the tendency for all positive dimensions of classroom experience—some more emotional, some motivational, and some learning- and achievement-oriented, to *vary together*. It is worth probing further the nature of such a construct, and how it should be conceptualized. Modern statistical techniques in which aspects of classroom life are broken into separate components or elements are very common. It may be somewhat less common to consider classroom experiences and interactions holistically, acknowledging that while various aspects may be distinguishable, they are also interrelated. It is important to consider the theoretical and practical implications of this interrelatedness.

Theoretically, a holistic perspective on classroom experiences is supported by phenomenological views, most especially flow theory. Flow is a theory of emergent or phenomelological motivation, one that has loaned itself to systematic investigation (Csikszentmihalyi and Csikszentmihalyi, 1988; Csikszentmihalyi, 1990). Few other constructs capture a state of “optimal experience” in which individuals simultaneously report a sense of challenge, relevance, effort, and concentration, as well as a sense of participation, belongingness and involvement toward clearly defined goals, together with positive emotional and motivational states. In such moments, individuals feel to be “firing on all cylinders.” Because these same experiences, in this study, were also significantly related to perceptions of perceived learning, and included the perception that instructional activities supported learning, they may be best characterized by a multidimensional view of student engagement as rooted in flow theory. With respect to practical implications, it may be that instructional modifications that improve one aspect of student classroom experience can have a more pervasive influence than might be realized. For example, researchers have found that relatively modest interventions in which students reflect on personal relevance of higher education course materials have surprisingly substantial effects on students' interest and achievement in a course (Harackiewicz et al., 2015). Much more needs to be known about how various aspects of course experience and achievement-related behaviors are interrelated.

In this study, the general construct of student engagement was strongly dispositional, varying more between students (69%) than within students (31%). A fundamental tenet of flow theory is that optimal experiences at the state level are cumulative, and their progressive accumulation can be a driving force in human development. A student who continually acts with curiosity and persistence when solving a problem in the accounting class over time may develop a trait for being curious and persistent relative to other students in the class. The same may be true for patterns of involvement and participation in class activities. Those who develop the pattern of experiencing flow have been described in terms of having an “autotelic personality” (Hektner, 1996) and developing psychological complexity (Csikszentmihalyi, 1993; Csikszentmihalyi and Rathunde, 1998) in which human potentialities are increasingly differentiated and integrated. Oppositely, repeated experiences of inaction, perceived debilitation, distraction, and the accompanying emotions of boredom or frustration, or what Csikszentmihalyi and Larson (1984) refer to collectively as “psychic entropy,” can develop into sustained disengagement or detachment. Further research should investigate the extent to which such a student-level construct is related to or characterized by other prevailing constructs such as learning or mastery goals orientation and competency beliefs (Ames, 1992; Butler, 2006; Urdan and Schoenfelder, 2006), self-regulated learning (Zimmerman, 2002; Cleary et al., 2012), or student learning patterns in higher education (Vermunt and Donche, 2017).

Current findings also suggest that factors explaining variation in students' classroom experiences beyond a general factor may be different at the within-student and between-student levels, which is not altogether surprising when one considers that the meanings of constructs that vary within students can be entirely different than those that vary between students. In this study, specific aspects of classroom experience explaining additional within-student variation in student survey responses beyond the general factor included intrinsic motivation, academic intensity, salience, and classroom self-esteem. At the between-student level, specific aspects of classroom experience included work orientation, learning orientation, classroom self-esteem, and disengagement. In addition to engagement, between-student variation in learning orientation also predicted both perceived learning and course grades. That is, students who consistently had clear goals for each class, regarded instruction as relevant and personally important, and believed that class activities were helpful also reported greater learning and earned higher grades in the course. This corroborates previous research demonstrating that students who are oriented toward the goals of learning and understanding, as opposed to other types of goals such as the desire to be recognized as competent or avoid appearing incompetent relative to others, are more likely to embrace challenge, exert effort, and succeed academically (Ames, 1992; Midgley, 2002).

Both the within-student Academic Intensity factor, and the between-student Work Orientation variable included skill, concentration, and effort (Academic Intensity also included challenge), as well as a negative emotionality (For Academic Intensity, positive loadings for detachment, irritation boredom, and learning interference; for Work Orientation, negative loadings for interest and enjoyment). Utilizing high level of skills, concentration and effort in challenging activities is central to flow experiences; consistent with flow theory, these experiences include positive affect and emotionality in the student engagement variable. Indeed, at the student level, interest and enjoyment are the highest loading items (β = 0.96 and β = 0.95, respectively) on student engagement, and yet they loaded negatively onto the Work Orientation factor. At the within-student level, it appears that there were some experiences in which the challenge of the instruction or activity was high and demanded a high level of effort. These experiences were sometimes joined with a sense of detachment or resignation—as when students become lost and are no longer able to follow the lesson or complete problems successfully. At the between-student level, it appears that some students may be characterized by high levels of concerted effort, but they are not truly interested in the material, and they do not enjoy it. In contrast to students characterized by the Engagement factor, this represents the profile of a “worker,” who is driven to achieve and be responsible, but takes no pleasure or joy in doing so (Csikszentmihalyi and Larson, 1984; Csikszentmihalyi and Schneider, 2000). It might be noted that the reliabilities of the academic intensity and work orientation variables were both unacceptably low (likely due to their inclusion of both “positive” and emotionally “negative” items), whereas the reliability of the engagement variable was excellent.

### Predictors and outcomes associated with student engagement and other aspects of classroom experience

A second goal of the study was to determine the degree to which general and specific dimensions of classroom experience were associated with classroom practices and perceptions, as well as student outcomes. Both within-students and between-student relationships were analyzed. Several student practices and perceptions had within-student effects on student engagement. For example, when students were taking notes, actively listening to the lecture, or working on problems (whether difficult or solvable problems), they reported being more engaged and paying more attention to instruction. Student engagement, in turn, was significantly related to perceived learning, and was shown to mediate an indirect relationship between these student practices and perceived learning. With respect to problem solving, we had expected that working on solvable problems would predict a higher quality of experience than difficult problems since the latter can be expected to produce anxiety, the state resulting when the challenge exceeds skill according to flow theory. However, results suggest that working on either type of problem was associated with greater engagement and attention. This result suggests that opportunity for action, even when the challenge exceeded students' skills, may be more engaging than when no such opportunity exists. Lack of a meaningful challenge and use of skills would be expected to produce a state of apathy, according to the theory (Csikszentmihalyi, 1990).

The negative within-person effect of taking notes, active listening, and working on both types of problems on intrinsic motivation was surprising. Again, it should be realized that these same student practices had positive effects on student engagement, which includes the same intrinsic motivation items (i.e., enjoyment, interest, and excitement). However, these negative relationships suggest that, in the context of the financial accounting class, practices such as taking notes and doing problems are frequently perceived as work activities, and could impede positive affect or states of greater relaxation relative to behaving more passively.

Students also reported lower levels of student engagement and attention when sitting in back of the class compared to when sitting in the front. Because this effect was significant both as a within-student and between-student effect, this may reflect a compromised quality of engagement when sitting toward the back of the large lecture hall, perhaps due to environmental differences such as an inferior ability to see and hear the instructor or greater distractions, as well as person-level or character attributes that may influence seat selection. Furthermore, there was a significant indirect effect of seating location on perceived learning and on grades as mediated by student engagement, at both the within-student and between-student levels. A previous, detailed study of the seating location issue from the same data set (Shernoff et al., 2017) utilizing a traditional factor structure also supported a relationship between the tendency to sit in the front of the class and course grades, with a variety of factors also acting as mediators. These factors included a learning orientation, classroom self-esteem, and intrinsic motivation. Because engagement was the only significant mediator when using the bifactor model, it appears as though the general engagement factor may subsume or encompass the mediating role of many factors in a traditional model. Findings also suggested that there is also a direct relationship between seating patterns and course grades, potentially related to differences in personality, achievement goals, learning patterns or other person-level factors.

### Implications for future research

Study findings are consistent with a growing trend in education and methodology research literatures rediscovering bifactor models as providing a better fit than non-bifactor models (e.g., Reise, 2013; Hamre et al., 2014; Wallace et al., 2016). In addition, the use of multilevel models allowed for a partitioning of variance of engagement and experience variables into within-student (e.g., engagement as a state) and between-student (i.e., engagement as a trait) components. As a generalization, the ICCs of the variables suggested approximately equal levels of within-student and between-student variability, and 69% of the variation in student engagement was average variation among students. This suggests that there was a context-specific as well as dispositional aspect to multiple aspects of classroom experience, and that the general factor of student engagement strongly dispositional.

We are not aware of any prior studies of student-reported engagement or experiences that have tested a bifactor model in a multilevel context. Taken together, findings from this study suggest that a bifactor approach can both have unique advantages to the traditional approach in data for which a general factor or strong unidimensional aspect exists. Among the greatest advantage of the bifactor approach is the enhanced abilities of interpretation stemming from the lack of multicollinearity. Thus, it may be of utility to other researchers when analyzing experience sampling data. It will be important for the utility of bifactor models to be replicated in future research.

### Implications for practice

Although many might consider the range of student learning strategies available to students in a large university lecture course to be constrained, the present study suggests that they can impact the quality of students' experiences as well as educational outcomes. Importantly, the present study suggests that active learning strategies and behaviors are a key facilitator of students' engagement, learning, and performance in the large lecture hall. Although much of the literature suggests that engagement is influenced by external and environmental factors (e.g., autonomy-supportive environments), the present study suggests that students' own choices and behaviors play an important role in bolstering their own engagement, even when the range of behavioral choices are limited. This phenomenon is most closely equated to what Reeve (2013) has refered to as *agentic engagement*. For example, students sitting in the front of the class, taking notes, actively listening, and working on problems appeared to create a higher quality learning experience for themselves. Thus, students might be encouraged to make decisions during instruction that increase their active involvement, including choice of seating, effortful listening, taking notes, and actively working on problems. Students who adopted such learning strategies in the present study reported greater engagement, which in turn predicted more positive student outcomes. On the instructional side, instructors should do what is possible to set up the learning environment and provide expectations for assuming an active posture toward learning, even in large lecture courses. For example, instructors can experiment with the use of small groups and/or increased monitoring (including TA monitoring) in order to support student learning goals.

### Study limitations

Results should be interpreted with caution in a number of respects. First, this was a correlational study, making inferences of causality and directionality speculative. Second, this study was also limited to two cohorts of a single undergraduate course in financial accounting occurring in a large, amphitheater-style classroom. Thus, results may not generalize to other types of educational settings, contexts, and subjects. Additional studies are needed.

Third, measures of student practices and perceptions, engagement, and perceived learning were based strictly on student self-report. Self-report measures of engagement have been found to range greatly in terms of their psychometric properties, and although they are necessary and have made significant contributions to our understanding, the use of multiple methods and measures is recommended by experts who have conducted systematic reviews of studies relying on self-reports of engagement (Fredricks and McColskey, 2012; Greene, 2015). The development of theory requires corroboration across studies and the use of multiple methods. Thus, similar work with multiple methods and corroboration with other indicators of experience such as diaries, notebooks, logs would be especially helpful in investigating the possibility of a general factor of classroom with greater implications for theory. We also acknowledge that perceived learning is not the same as an “objective” measure of content learning such as test scores. It would be useful for future work to additionally utilize performance-based measures of learning beyond grades.

Finally, there has been a general lack of consensus in engagement factors, and thus we acknowledge that others could disagree with the composition or labeling of factors. Also, the factor structure in this study might not generalize to other populations or research designs.

## Conclusion

In sum, this study suggests the feasibility and potential value of conceptual and analytic approaches elucidating both general and specific elements of classroom experience. In this study, a two-level bifactor model offered both greater feasibility and better fit to the data than traditional (i.e., non-bifactor) factorial structures. This suggested a significant unidimensionality to multiple aspects (e.g., perceptual, emotional, motivational, and cognitive) of students' experience in a large, undergraduate accounting course. In terms of within-student effects, sitting in the front of the class (compared to sitting in the back), taking notes and working on both solvable and difficult problems during class had a positive effect on student engagement and attention, which in turn predicted perceived learning. With respect to between-student effects, the tendency to sit in front seats had a significant effect on student engagement, which in turn had a significant effect on course grades. Findings were suggestive of a strong mediating role of student engagement in the relationship between student practices and learning strategies on educational outcomes such as learning and achievement. While these results have implications for theory, research, and practice, further studies are needed to corroborate results and provide additional clarifications before firm conclusions can be drawn.

## Ethics statement

This study was carried out in accordance with the recommendations of Rutgers University Full IRB Review Board with written informed consent from all subjects. All subjects gave written informed consent in accordance with the Declaration of Helsinki. The protocol was approved by the Rutgers University IRB Review Board.

## Author contributions

DS contributed to the conception and design of the study, and led analyses, writing of the manuscript, interpretation, and revision. ER contributed to analyses and writing of the manuscript. AS contributed to the conception and design of the study, instructed the course providing the context for the study, and contributed to the writing of the manuscript. RS contributed to the conception and design of the study, and to the writing of the manuscript. LS contributed to the conception and design of the work, and led data collection. DB contributed to the interpretation of results and to the revising of the manuscript. All authors provided approval of the final submitted version of the manuscript and agree to be accountable for all aspects of the work in ensuring that questions related to the accuracy or integrity of any part of the work are appropriately investigated and resolved.

### Conflict of interest statement

The authors declare that the research was conducted in the absence of any commercial or financial relationships that could be construed as a potential conflict of interest.
